# Transcriptomics and metabolomics reveal functional nanoplastics-induced male reproductive damage and resveratrol antagonistic effects

**DOI:** 10.1186/s12951-026-04283-8

**Published:** 2026-03-16

**Authors:** Fang Zhang, Nenghua Zhang, Chunji Wang, Li Zhang, Yujia Yang, Yue Jia, Xiaowen Huang, Minghui Li, Jie Tang, Long Xu

**Affiliations:** 1 College of Biological, Chemical Science and Engineering, Forensic and Pathology Laboratory, College of Medicine, Jiaxing University, Jiaxing, 314001 ZJ China; 2 Central Laboratory of Jiaxing Hospital of Traditional Chinese Medicine, Jiaxing University, Jiaxing, 314001 ZJ China; 3 College of Life Sciences and Medicine, Zhejiang Sci-Tech University, Hangzhou, 310053 ZJ China; 4 School of Life Sciences, Zhejiang Chinese Medical University, Hangzhou, 310053 ZJ China; 5 School of Bioengineering, Chongqing University, Chongqing, 400045 China

**Keywords:** Polystyrene nanoparticles, Functionalized polystyrene nanoparticles, Transcriptomics, Metabolomics, Reproductive damage, Resveratrol

## Abstract

Polystyrene nanoparticles (PS-NPs) can undergo surface functionalization under environmental conditions, and their accumulation in living organisms may pose a potential reproductive toxicity risk to males, though the underlying mechanisms remain incompletely understood. This study employed integrated transcriptomic and metabolomic analyses to investigate the molecular mechanisms of testicular injury in male ICR mice induced by a 28-day exposure to plain PS-NPs (PS), amino-modified PS-NPs (PS-NH₂), and carboxyl-modified PS-NPs (PS-COOH) (0.08 μm; 50 mg/kg/day), and to evaluate the concurrent protective effects of resveratrol (RES; 100 mg/kg/day). Results showed that PS-NH₂ and PS-COOH caused severe spermatogenic impairment concurrent with blood-testis barrier disruption and inflammation, marked by mislocalization of ZO-1, occludin, and vimentin, and an imbalance between upregulated TNF-α and downregulated IL-10. RES treatment effectively alleviated these alterations. Transcriptomic analysis identified 1633 differentially expressed genes, primarily associated with lipid metabolism, spermatogenesis, and apoptosis. KEGG enrichment indicated significant involvement of the PI3K-AKT signaling pathway. Metabolomic profiling identified 158 differentially expressed metabolites, linked to pathways such as pyrimidine metabolism, bile secretion, and cholesterol metabolism. Integrated multi-omics analysis suggested that different types of PS-NPs affected distinct biological pathways: the PS group was mainly associated with taurine metabolism, the PS-NH₂ group with bile secretion and cholesterol metabolism, and the PS-COOH group with bile secretion and insulin resistance. RT-qPCR analyses confirmed that PS-NPs significantly upregulated the mRNA expression of inflammatory markers (*TNF-α*, *IL-10*) and pro-apoptotic factors (*BAX*,* caspase-3*), alongside a downregulation of *PI3K*, *AKT*, *caspase-9*, and *caspase-8*. Increased protein levels of p-AKT, PI3K, the BAX/BCL-2 ratio, and cleaved caspase-3 were observed, concomitant with decreased levels of caspase-3, caspase-9, and caspase-8. These effects were partially ameliorated by RES treatment. Our data indicate that functionalized PS-NPs induce severe testicular damage, potentially via activation of the PI3K-AKT pathway, alongside induced inflammation and apoptosis. Metabolic disturbances such as those in bile secretion may be contributing factors. Importantly, RES treatment effectively mitigates these adverse effects. Collectively, our study elucidates a key mechanism underlying PS-NPs reproductive toxicity and proposes a promising therapeutic intervention with RES.

**Graphical abstract:**

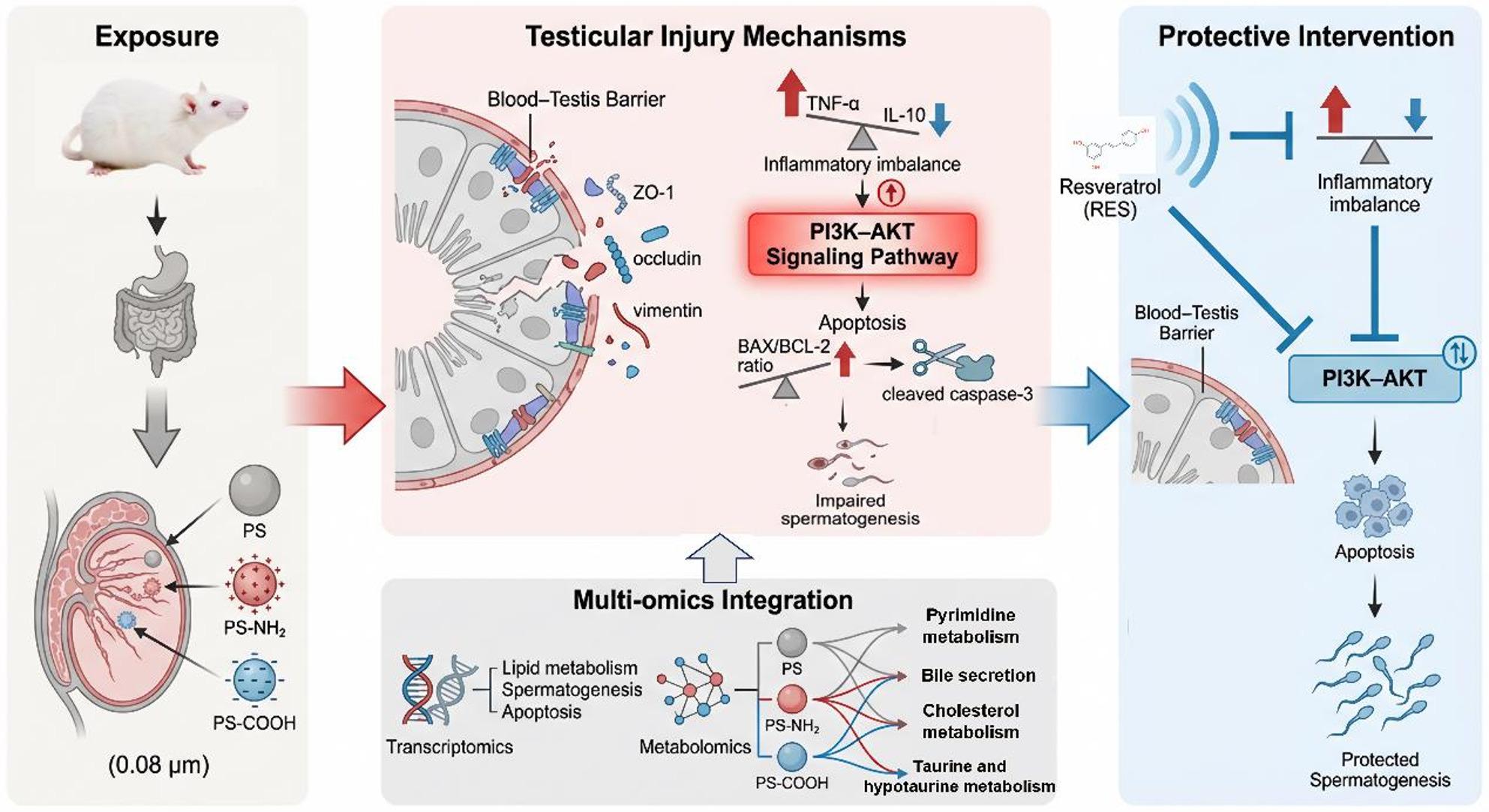

**Supplementary Information:**

The online version contains supplementary material available at 10.1186/s12951-026-04283-8.

## Introduction

Plastic pollution is widely recognized as one of the most pressing global environmental issues. Among these pollutants, micro- and nanoplastics (MNPs) are ubiquitous in water, soil, and air [1]. MNPs can enter the human body through multiple pathways, including inhalation, dietary intake, and dermal contact [2]. They have been detected in various human tissues and bodily fluids such as urine, blood, and placenta, likely through food chain accumulation [3]. Accumulating evidence indicates that MNPs exert toxic effects on multiple systems, including the digestive, respiratory, cardiovascular, nervous, reproductive, immune, and endocrine systems [4].

In recent years, concerns regarding male reproductive health have grown considerably. A comprehensive analysis of global sperm count trends reveals a significant decline in sperm concentration and total sperm count among men in Western countries over the past few decades [5]. Such reproductive impairments not only affect individual fertility but may also pose long-term risks to demographic stability and public health systems. Among various environmental triggers, the reproductive toxicity of persistent pollutants has drawn increasing attention. Due to their pervasive distribution and bioaccumulative potential, MNPs are regarded as potentially high-risk factors. Clinical studies have detected polyethylene, polystyrene, and polyvinyl chloride in human semen samples, demonstrating that polystyrene nanoplastics (PS-NPs) can directly reach the male reproductive system [6]. For instance, exposure of male mice to PS-NPs (25–100 nm) at a dose of 50 mg/kg (within a range of 0.5–144 mg/kg) significantly impaired fertility. The manifestations encompassed testicular histopathological alterations, such as seminiferous tubule dilation and diminished intraluminal sperm counts, alongside a general deterioration in sperm quality. Mechanistic investigations revealed that these effects were attributable not only to PS-NPs-induced testicular apoptosis and inflammatory responses but also to a direct suppression of sperm capacitation, mediated by enhanced ubiquitination of Ras-related C3 botulinum toxin substrate 1 (RAC1) and cell division control protein 42 (CDC42) [7]. These findings underscore the potential threat of PS-NPs to male reproductive health and highlight the importance of investigating their reproductive toxicity and possible interventions.

Under environmental conditions, microplastics undergo fragmentation into nanoplastics (NPs) via ultraviolet (UV) radiation and oxidation. This degradation process is often accompanied by reactive oxygen species (ROS) generation and changes in the surface chemical properties of nanoplastics, including the introduction of oxygen-containing functional groups [8]. As a result, environmentally relevant PS-NPs often exhibit diverse surface characteristics rather than a uniform composition. Studies have shown that surface chemistry influences PS-NPs toxicity. For instance, compared to that of plain PS-NPs (PS), amino-modified PS-NPs (PS-NH₂) exhibited the most significant inhibition of antioxidant enzymes in gills and mantle, while carboxyl-modified PS-NPs (PS-COOH) affect oxidative stress indices [9]. In human liver cancer HepG2 cells, surface-functionalized PS-NPs presented greater cytotoxicity than PS, as shown by a more pronounced decrease in cell viability [10]. Distinct foliar absorption and translocation are observed for PS-NPs with different surface charges. For example, PS-NH₂ penetrated more deeply into the leaves and disperses uniformly within the mesophyll cells, leading to more pronounced physiological effects [11]. To date, most toxicological studies on modified PS-NPs have focused on aquatic organisms, cell cultures, and plants. Although reproductive impairments induced by unmodified NPs have been reported in zebrafish, oysters, *Caenorhabditis elegans*, chickens, mice, and rats [12], few studies have addressed the reproductive toxicity of surface-functionalized PS-NPs in mammals, and the underlying mechanisms remain poorly understood. Therefore, systematic investigation into the reproductive toxicity of differentially functionalized PS-NPs in mammalian models is of considerable significance.

Current research on the reproductive toxicity of PS-NPs relies heavily on single-omics approaches. For example, transcriptomic analyses have revealed that PS-NPs (80 nm) at doses of 0, 3, 6, and 12 mg/kg/day compromises blood-testis barrier integrity in rats by triggering inflammatory responses and apoptosis. This damage reduces the localization of junctional proteins, disrupts the spermatogenic microenvironment, and ultimately leads to reduced sperm count and motility [13]. Metabolomics-based studies have elucidated that exposure to 50 nm PS-NPs at 3 mg/mL/day or 90 nm PS-NPs at 15 mg/mL/day induces male reproductive toxicity by disrupting the gut microbiota-metabolite axis [14]. Multi-omics strategies, which integrate data from genomics, transcriptomics, proteomics, and metabolomics, provide a more comprehensive understanding of biological responses [15]. For instance, a previous study employing transcriptomic and proteomic analyses demonstrated that exposure to PS-NPs of varying sizes (25, 50, and 100 nm) at a consistent concentration of 5 mg/mL (0.2 mL administration) interfered with spermatogenesis by altering gene and protein expression, promoting testicular cell apoptosis, and impairing tight junctions [16]. In another study, using integrated metabolomic and proteomic analysis, Liu et al. [17] found that 60 nm PS-NPs at 20 mg/kg/day impaired sperm motility and fertilization rates via triggering testicular immune-inflammatory responses and inducing spermatic metabolic reprogramming, which disrupted key cellular pathways. Nevertheless, multi-omics studies on the male reproductive toxicity of functionalized PS-NPs remain scarce. This study applies integrated transcriptomic and metabolomic sequencing to establish a direct link between gene expression and metabolic phenotypes, thereby systematically evaluating the effects of PS, PS-NH₂, and PS-COOH on testicular morphology, sperm quality, and blood-testis barrier integrity.

Several natural compounds, such as N-acetylcysteine [18], curcumin [19], and rhamnetin [20], have shown protective effects against PS-NPs-induced reproductive damage. Resveratrol (RES), a natural polyphenol present in grapes, blueberries, and other plants [21], has gained attention for its potent antioxidant and anti-inflammatory properties [22]. Importantly, RES has been shown to ameliorate male reproductive injury caused by various pollutants, including bisphenol A [23] and trichloropropanol [24], suggesting its capacity for multi-target intervention. However, whether RES can specifically counteract PS-NPs-induced reproductive toxicity remains unknown. Therefore, this study introduces RES as a promising therapeutic candidate and systematically evaluates its efficacy in alleviating PS-NPs-induced reproductive damage.

## Materials and methods

### Materials

PS, PS-COOH, and PS-NH₂ were purchased from Tianjin Baseline ChromTech Co., Ltd. (Tianjin, China). All nanoparticles were characterized as microspheres with an average particle size of 80 nm and a solid content of 2.5% (w/v) and exhibited good dispersion stability. RES (CAS: 501-36-0, purity > 99%) was purchased from Aladdin (Shanghai, China). The Hematoxylin and Eosin (H&E) Staining Kit (Cat# C0105S) was purchased from Beyotime Co., Ltd. (Shanghai, China). The Alcian Blue-Periodic Acid Schiff (AB-PAS) Staining Kit (Cat# G1285) was purchased from Solarbio Science & Technology Co., Ltd. (Beijing, China).

### Characterization of three types of PS-NPs

The morphology and particle size of PS, PS-COOH, and PS-NH₂ were examined using scanning electron microscopy (SEM; ZEISS Sigma 360, Germany). Surface functional groups were characterized by Fourier transform infrared spectroscopy (FTIR; Nicolet iS20, USA). The hydrodynamic diameter distribution and zeta potential of the nanoparticles in aqueous suspension were determined using a dynamic light scattering (DLS) (Malvern Zetasizer Pro, UK).

### Animal models

A total of 40 specific pathogen-free (SPF) male ICR mice (7 weeks old, weighing 40 ± 0.4 g) were obtained from the Experimental Animal Center of Jiaxing University. All animals were housed under controlled barrier conditions: temperature 24 ± 2 °C, relative humidity 40–60%, and a 12 h / 12 h light-dark cycle, with free access to food and water. The experimental procedures were approved by the Animal Ethics Committee of Jiaxing University (Approval No. JUMC2021-052). After one week of acclimatization, the mice were randomly assigned to eight groups (*n* = 5 per group) based on body weight: Control, PS, PS-NH₂, PS-COOH, RES, RES + PS, RES + PS-NH₂, and RES + PS-COOH. Animals were co-treated with PS-NPs (50 mg/kg/day) and RES (100 mg/kg/day) administered by oral gavage [23]. With reference to published data, the estimated daily intake of MNPs in adults ranges from 0.238 to 11.9 mg/kg, corresponding to a mouse equivalent dose of approximately 2.93–146 mg/kg [25]. Based on the experimental design, a dose of 50 mg/kg was selected for PS-NPs exposure and administered daily by oral gavage. In this study, RES was administered at a dose of 100 mg/kg. This dosage was selected based on existing animal studies, which indicate that a daily dose ranging from 5 to 100 mg/kg is required to elicit specific biological effects. Consequently, the 100 mg/kg dose employed herein is at the upper end of this efficacious range, thereby aiming to ensure a pronounced intervention effect [26]. Control animals and the RES-alone group received an equal volume of ultrapure water by gavage. Body weight was recorded weekly throughout the study. After 4 weeks of intervention, all mice were euthanized under anesthesia. Testes and epididymides were collected and weighed for subsequent analysis.

### Sperm count and morphology analysis

For sperm count, the cauda epididymis was incised with fine scissors and incubated in pre-warmed physiological saline for approximately 15 min to allow for the natural swim-out of sperm. The pellet was resuspended in fixative (0.5% NaHCO₃, 1% formaldehyde) and fixed for 2 h at room temperature. Finally, sperm count was determined using a hemocytometer [27].

For sperm morphology analysis, the epididymis was cut with scissors in 1 × PBS and incubated at 37 °C for 15 min to release sperm. After filtration, smears were prepared, air-dried, fixed with methanol, and stained with 1% eosin. For each sample, ten random fields of view were examined under an upright microscope (10 × magnification; BX53 Olympus, Japan) to assess sperm morphology, with a minimum of 500 spermatozoa counted per sample for the determination of the abnormality rate.

### H&E Staining

Testicular tissues were fixed in 4% paraformaldehyde (Solarbio; Cat# P1110), embedded in paraffin, and sectioned. The sections were then subjected to H&E staining according to a standard protocol as described previously [28]. Morphological changes in the testicular tissue were evaluated using an upright light microscope (BX53 Olympus, Japan).

### AB-PAS staining

The paraffin sections were processed for AB-PAS staining according to standard protocols as described previously [29]. Acrosomal structures were examined using an upright light microscope (BX53 Olympus, Japan).

### Immunohistochemistry

Immunohistochemical analysis was performed on paraffin-embedded testicular sections as previously described, with minor modifications [29]. In brief, sections were deparaffinized, rehydrated, and subjected to antigen retrieval in citrate buffer (pH 6.0). After blocking endogenous peroxidase activity and nonspecific binding sites, the sections were incubated overnight at 4 °C with primary antibodies against zonula occludens-1 (ZO-1; Cat# 21773-1-AP, Proteintech), occludin (Cat# 27260-1-AP, Proteintech), vimentin (Cat# MAB-0735, Fuzhou Maixin), tumor necrosis factor-alpha (TNF-α; Cat# PAA133Mu01, Cloud-Clone Corp), and interleukin-10 (IL-10; Cat# PAA056Mu01, Cloud-Clone Corp). This was followed by incubation with an HRP-conjugated secondary antibody (Cat# K5007, Dako) at room temperature. Signal detection was carried out using 3,3-diaminobenzidine (DAB), and the sections were counterstained with hematoxylin, dehydrated, cleared, and mounted. Morphological changes in the testicular tissue were evaluated under an upright light microscope (BX53 Olympus, Japan).

### Transcriptomic analysis

This study included five biological replicates per group: Control group (*n* = 5), PS group (*n* = 5), PS‑COOH group (*n* = 5), and PS‑NH₂ group (*n* = 5). Total RNA was extracted from testicular samples using TRIzol reagent (Invitrogen, USA) according to the manufacturer’s protocol. After RNA extraction, all samples were assessed for concentration and purity using a NanoDrop ND‑1000 spectrophotometer (NanoDrop, Wilmington, DE, USA). RNA integrity was further evaluated with a Bioanalyzer 2100 system (Agilent, CA, USA). Samples were only used for library preparation if they simultaneously met the following criteria: RNA concentration > 50 ng/µL, RNA integrity number (RIN) > 7.0, and total RNA amount > 1 µg. Reverse-transcribed to construct cDNA libraries with an average insert size of 300 ± 50 bp. Paired-end sequencing (150 bp) was performed on an Illumina NovaSeq™ 6000 platform. Genes with | log₂ (fold change) | ≥ 1 (i.e., fold change ≥ 2) and *P*‑value < 0.05 were considered differentially expressed. Transcriptome sequencing was performed by LC-Bio Technologies Co., Ltd. (Hangzhou, China).

### Untargeted metabolomic analysis

Tissues were thawed on ice, and approximately 100 mg of tissue were homogenized in 1 mL of pre-cooled extraction buffer (isopropanol: acetonitrile: water, 2:1:1, v/v/v). The mixture was vortexed for 1 min, incubated at room temperature for 10 min, and stored at −20℃ overnight. After centrifugation at 4000 × g for 20 min at 4℃, the supernatant was collected and transferred to a 96-well plate. Metabolite profiling was performed using an ultra-high-performance liquid chromatography-high-resolution mass spectrometry (UHPLC-HRMS) system. Detailed instrument parameters are provided in Supplementary Note 1. Quality control (QC) samples were prepared by pooling equal volumes of all experimental samples. QC injections were evenly distributed at the beginning, middle, and end of the sample sequence to monitor instrumental performance. The reproducibility among QC samples was evaluated using Pearson correlation analysis, and the results are presented in Supplementary Fig. 1. Subsequent quality control and data processing were conducted using the metaX software: low‑quality peaks were first removed (missing in > 50% of QC samples or > 80% of actual samples), followed by median normalization for data scaling, and finally minimum value imputation for missing value filling. Initial identification was carried out using the open‑source metaX software by matching the exact mass (m/z) against public databases such as the human metabolome database (HMDB) and the kyoto encyclopedia of genes and genomes (KEGG). To improve confidence, we utilized a proprietary in‑house MS/MS spectral library established by LC‑Bio (Hangzhou, China). The experimental MS² spectra were matched against this curated library, yielding metabolite identifications with higher reliability. The differential expression criteria for metabolites were set as | log₂ (fold change) | ≥ log₂ (1.2) (i.e., fold change ≥ 1.2), *P*‑value < 0.05 and VIP ≥ 1. Metabolome analysis was performed by (LC-Bio Technologies Co., Ltd., Hangzhou, China).

### Integrated analysis of transcriptomics and metabolomics

During the differential screening step, for transcriptomics (based on RNA‑seq data), the criteria for identifying differentially expressed genes (DEGs) were set as an adjusted *q*‑value < 0.05 and | log₂ (fold change) | ≥ 1; for metabolomics, the criteria for differentially abundant metabolites (DAMs) were *P*‑value < 0.05, VIP ≥ 1, and | log₂ (fold change) | ≥ log₂(1.2). KEGG pathway enrichment analyses were performed separately for DEGs and differential metabolites, and pathways enriched in both groups were merged and visualized via a bar chart to show the co‑enriched pathways along with the numbers of genes and metabolites involved. To further explore the coordinated regulatory relationships between genes and metabolites within these pathways, a metabolite‑gene‑pathway interaction network was constructed, where nodes represent metabolites or genes, and edges represent their co‑occurrence or regulatory relationships within the same pathway.

### RT-qPCR

Total RNA was extracted using the MiniBEST Universal RNA Extraction Kit (TaKaRa; Cat# 9767, Japan). Genomic DNA was removed, and cDNA was synthesized from 1 µg of total RNA using the PrimeScript™ RT Reagent Kit with gDNA Eraser (TaKaRa; Cat# RR047A, Japan). qPCR was performed using TB Green^®^ Premix Ex Taq™ II (TaKaRa; Cat# RR820A, Japan) on a QuantStudio 3 Real-Time PCR System (Thermo Fisher Scientific, USA). The following genes were analyzed: *PI3K*, *AKT*, *TNF-α*,* IL-10*, *BAX*, *BCL-2*, *caspase-3*, *caspase-8* and *caspase-9*. The thermal cycling conditions were: 95℃ for 30 s, followed by 40 cycles of 95℃ for 5 s and 60℃ for 15 s. β-actin was used as the reference gene, and relative expression levels were calculated using the 2^ ^(−ΔΔCt)^ method. The primers utilized in our study were presented in Supplementary Table 1.

### Western blotting

Testicular tissues were homogenized in RIPA lysis buffer (Cat# 89902, Thermo Fisher, USA) containing protease inhibitors and sonicated on ice. Lysates were centrifuged at 12,000 rpm for 5 min at 4℃, and the supernatant was collected. Protein concentration was determined using a Bicinchoninic acid assay (BCA) Protein Assay Kit (Cat# P0012, Beyotime, China). Equal amounts of protein (60 µg) were separated by SDS-PAGE and transferred to polyvinylidene difluoride membrane (PVDF; Bio-Rad, Hercules, USA). After blocking with 5% non-fat milk(Cat# 9999, CST, USA) in TBST, membranes were incubated overnight at 4℃ with the following antibodies : against BCL2-associated X protein antibody (BAX; Cat# 2772, CST, USA), B-cell lymphoma 2 antibody (BCL-2; Cat# ab32124, Abcam, UK), cysteine aspartic acid-specific protease-3 antibody (caspase-3; Cat# 9662, CST, USA), cleaved caspase-3 antibody (Cat# 9664, CST, USA), cysteine aspartic acid-specific protease-8 antibody (caspase-8; Cat# 4790, CST, USA), cysteine aspartic acid-specific protease-9 antibody (caspase-9; Cat# 9508, CST, USA) AKT serine/threonine kinase antibody (AKT; Cat# 4685, CST, USA), phosphorylated AKT antibody (p-AKT; Cat# 66444-1-Ig, Proteintech, China), phosphatidylinositol 3-kinase antibody (PI3K; Cat# A00318-1, Boster, China), and β-actin antibody (Cat# 66009-1-Ig, Proteintech, China). Membranes were then incubated with HRP-conjugated secondary antibodies for 1 h at room temperature. Protein bands were measured via Bio-Rad ChemiDoc MP system (Bio-Rad, Hercules, USA) and band intensities were quantified with ImageJ software (Maryland, USA) after normalization to β-actin.

### Statistical analysis

All data are presented as mean ± SD. Statistical analyses were performed using GraphPad Prism 9.0 (San Diego, California, USA) and SPSS 22.0 (IBM, Chicago, USA). One-way ANOVA was used for multiple group comparisons, followed by Tukey’s post hoc test for pairwise comparisons. A two-sided *P*-value of < 0.05 or < 0.01 was considered statistically significant.

## Results

### Characterization of PS, PS-COOH, and PS-NH₂

**Fig. 1:**
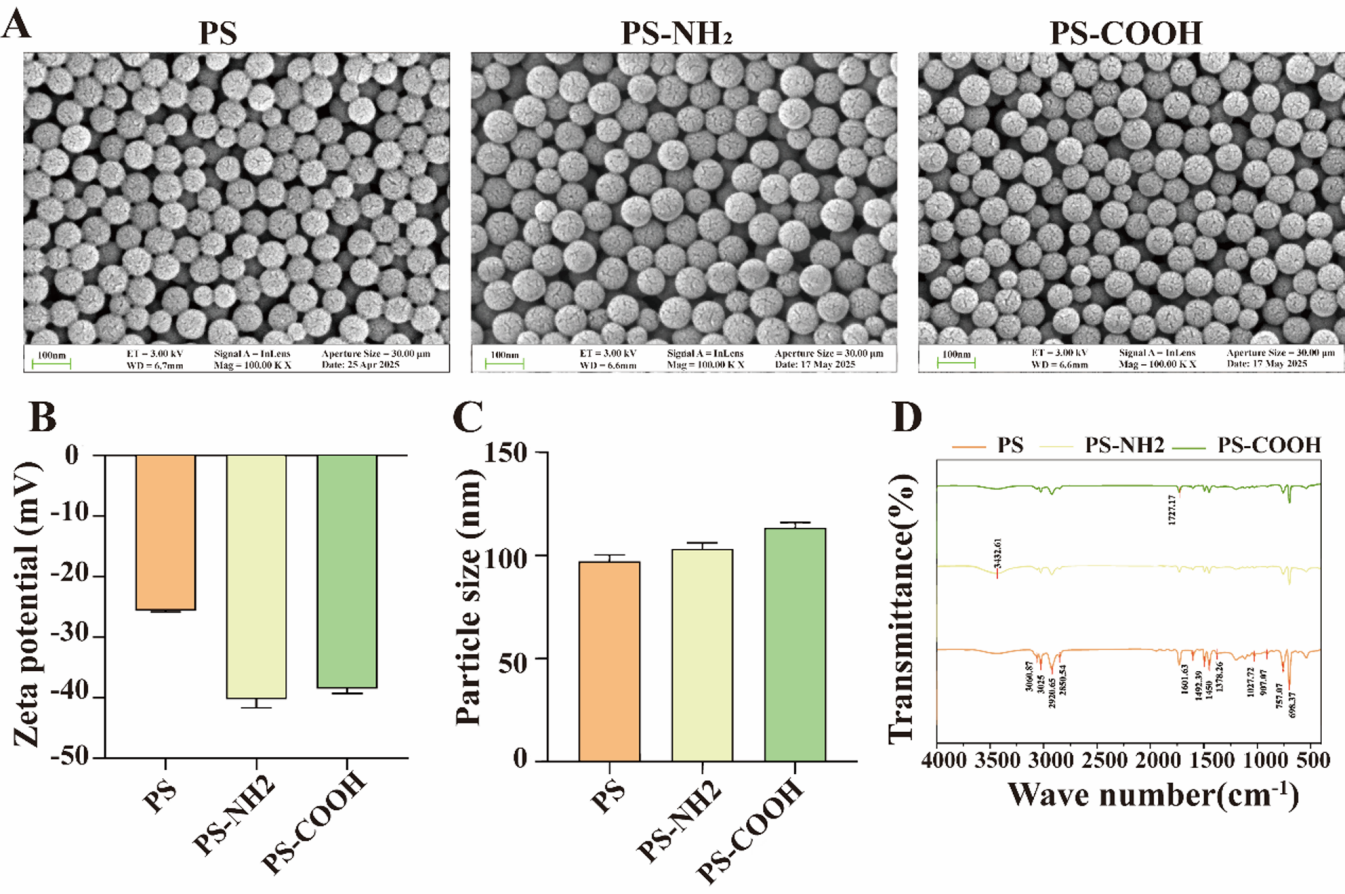
Characterization of plain PS-NPs (PS), amino-modified PS-NPs (PS-NH₂), and carboxyl-modified PS-NPs (PS-COOH). (**A**) Representative scanning electron microscopy (SEM) images of PS, PS-NH₂, and PS-COOH nanoparticles (scale bar = 100 nm). (**B**) Zeta potential values of the three PS-NPs in aqueous solution. (**C**) Hydrodynamic particle size of the three PS-NPs in aqueous solution measured by dynamic light scattering (DLS). (**D**) Fourier transform infrared (FTIR) spectra showing the characteristic chemical bonds of each type of PS-NPs

SEM analysis revealed that all three PS-NPs exhibited regular spherical morphology with a uniform size distribution, indicating good monodispersity **(**Fig. 1A**)**. Zeta potential measurements demonstrated that all samples carried negative surface charges, with values of −25.62 ± 0.24 mV for PS, −40.22 ± 1.48 mV for PS-NH₂, and −38.48 ± 0.81 mV for PS-COOH, respectively **(**Fig. 1B**)**. In Milli-Q water, the PS, PS-NH₂, and PS-COOH nanoparticles exhibited good monodispersity with average hydrodynamic diameters of 97.01 ± 2.59 nm, 103.24 ± 2.35 nm, and 113.45 ± 2.26 nm, respectively. Their corresponding low polydispersity index (PDI) values of 0.035, 0.047, and 0.097 further confirmed the colloidal stability of the dispersions **(**Fig. 1C**)**. The hydrodynamic diameters determined by DLS exceeded the manufacturer’s specifications. This is a common observation, primarily due to the fact that DLS measures the particle size including its hydration layer and reports an intensity-weighted average, which is sensitive to the presence of larger aggregates or particles [30, 31]. FTIR spectroscopy analysis showed generally similar absorption profiles among the three particle types **(**Fig. 1D**)**. Characteristic absorption peaks at 907.07 cm^-^¹ and 1027.72 cm^-^¹ were attributed to in-plane bending vibrations of C-H bonds in the benzene rings. Additionally, PS-COOH displayed a distinct peak at 1727.17 cm^-^¹, corresponding to C = O stretching vibrations of the carboxyl group, while PS-NH₂ showed a broad, enhanced absorption at 3432.61 cm^-^¹, consistent with N-H stretching vibrations of the amine group. These results collectively confirm the successful synthesis and surface functionalization of the nanoparticles, indicating good material stability suitable for subsequent toxicological experiments.

### Functionalized NPs induced severer testicular and sperm histopathology, and the antagonistic effect of RES

**Fig. 2:**
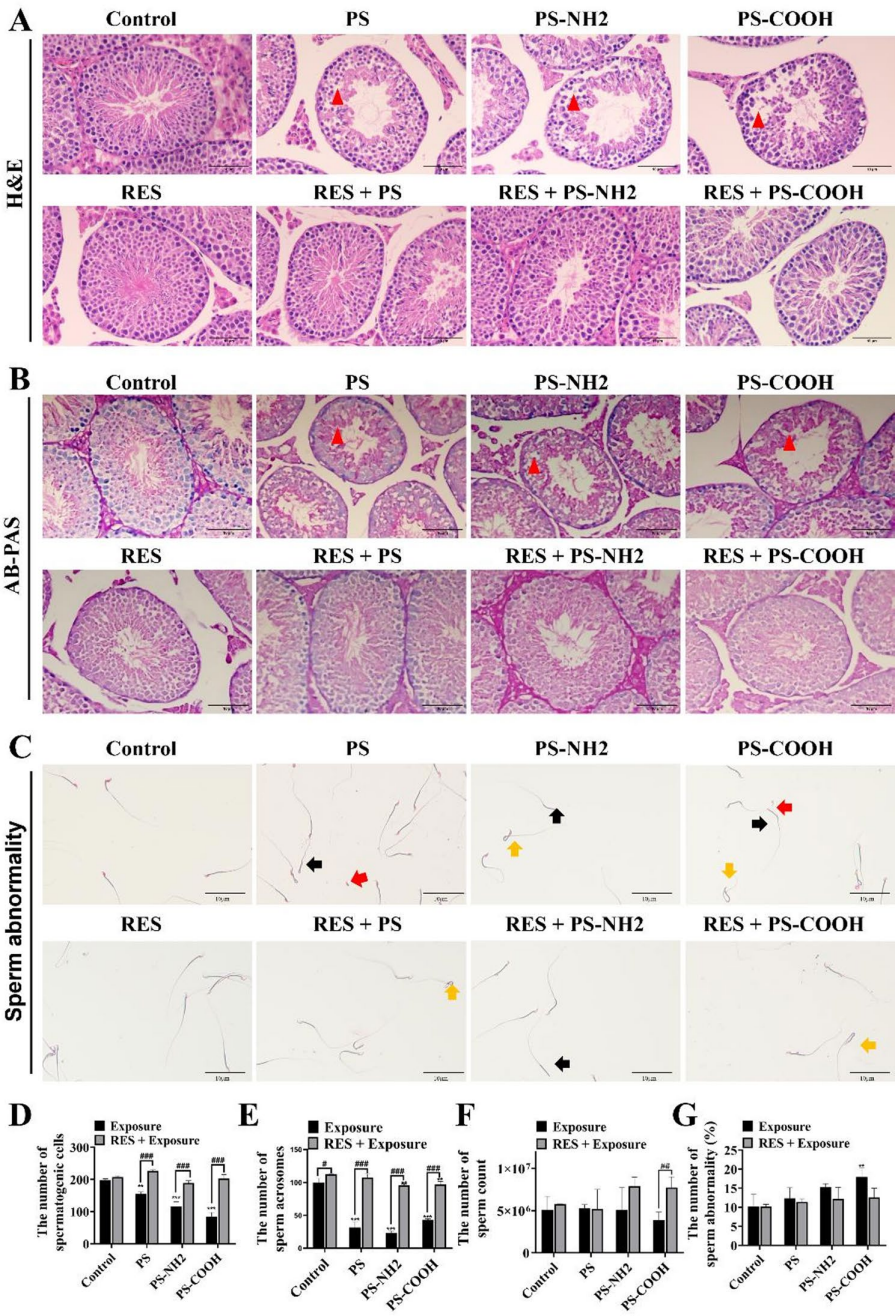
PS-NPs induced testicular and sperm damage in ICR mice. (**A**) Representative images of Hematoxylin and Eosin (H&E)-stained testicular sections showing the histopathological changes induced by PS-NPs (scale bar = 10 μm). (**B**) Representative images of Alcian Blue-Periodic Acid Schiff (AB-PAS)-stained testicular sections showing the status of acrosome formation in spermatids (scale bar = 10 μm). (**C**) Representative images of sperm morphology. Arrows indicate different deformity types: red (head), black (tail), and yellow (neck) (scale bar = 10 μm). (**D**) Quantification of spermatogenic cells per seminiferous tubule. (**E**) Quantification of sperm acrosomes. (**F**) Sperm count. (**G**) Sperm abnormality rate. Data are presented as the mean ± SD (*n* = 5); statistical significance is shown as follows: ^*^ for comparisons between the exposed group and the control group (^*^P < 0.05, ^**^P < 0.01, ^***^P < 0.001); ^#^ for comparisons between the exposed group and the antagonistic group (^#^*P* < 0.05, ^##^*P* < 0.01, ^###^*P* < 0.001)

Compared to the control group, all exposure groups displayed varying degrees of spermatogenic impairment, characterized by thinning of the germinal epithelium, disorganized and loosely arranged cells, and a marked reduction in luminal sperm **(**Fig. 2A**)**. AB-PAS staining indicated fewer acrosome-intact spermatozoa in exposed mice compared with the control group **(**Fig. 2B**)**. Abnormal sperm morphological changes including head defects, tail truncations, and neck folding were found **(**Fig. 2C**)**. PS-NPs exposure significantly reduced the numbers of spermatogenic cells, acrosomal cells, and total sperm, along with an elevated sperm abnormality rate, with PS-COOH and PS-NH₂ provoking more severe damage **(**Figs. 2D-G**)**. Importantly, RES co-treatment markedly alleviated these histopathological alterations and sperm parameter abnormalities.

### RES alleviated PS-NPs-induced blood-testis barrier (BTB) damage and inflammation

**Fig. 3:**
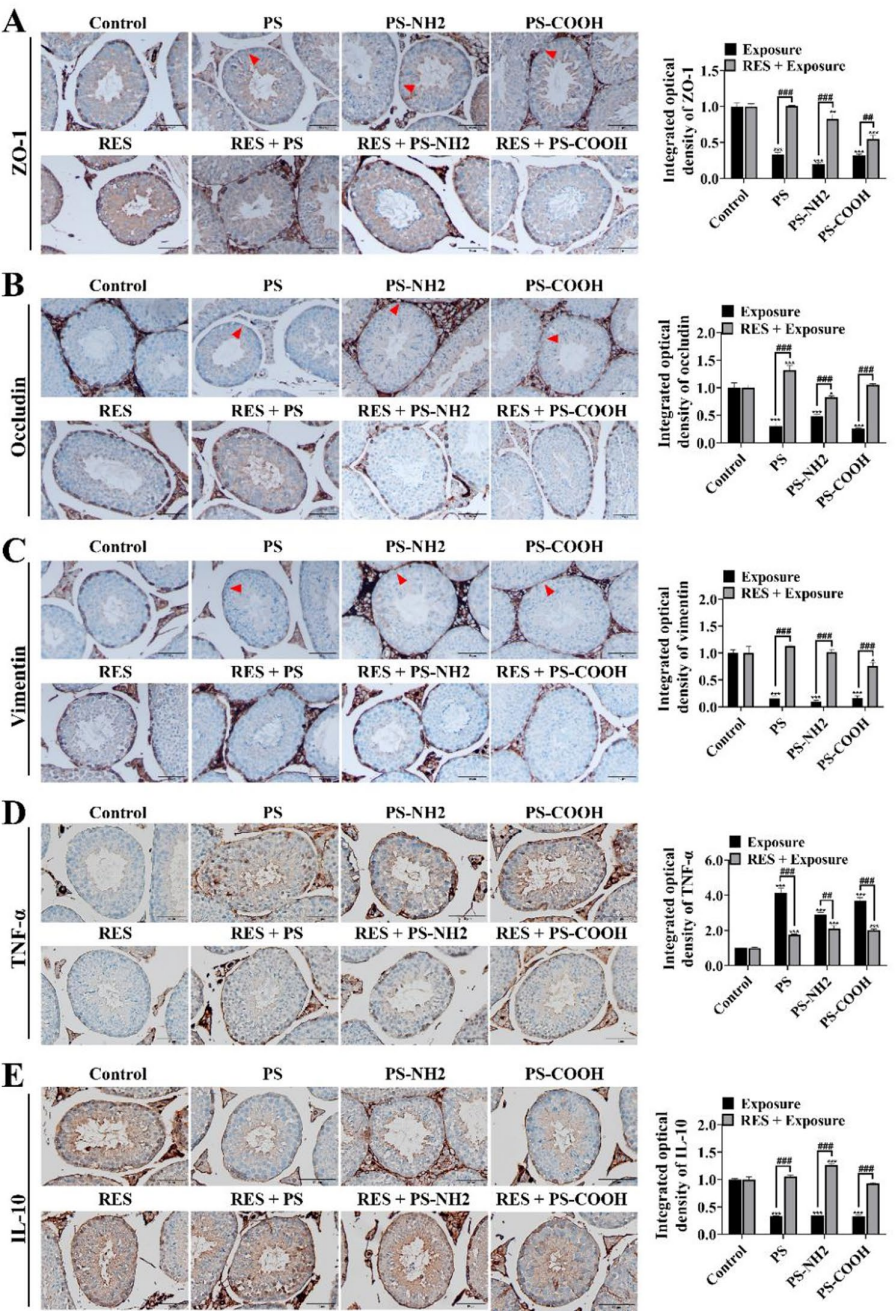
PS-NPs compromised the blood-testis barrier integrity and triggered testicular inflammation in ICR mice. (**A**) Representative immunohistochemistry images of ZO-1 in the testis, showing its disrupted expression following PS-NPs exposure (scale bar = 10 μm). (**B**) Representative immunohistochemistry images of occludin in the testis, showing its altered distribution after PS-NPs exposure (scale bar = 10 μm). (**C**) Representative immunohistochemistry images of vimentin in Sertoli cells, displaying cytoskeletal disorganization (scale bar = 10 μm). (**D**) Representative immunohistochemistry images showing the upregulated expression of the pro-inflammatory factor TNF-α (scale bar = 10 μm). (**E**) Representative immunohistochemistry images showing the expression changes of the anti-inflammatory factor IL-10 (scale bar = 10 μm). Data are presented as the mean ± SD (*n* = 5); statistical significance is indicated as follows: ^*^ for comparisons between the exposed group and the control group (^*^P < 0.05, ^**^P < 0.01, ^***^P < 0.001); ^#^ for comparisons between the exposed group and the antagonistic group (^#^*P* < 0.05, ^##^*P* < 0.01, ^###^*P* < 0.001)

Compared to the control group, all exposed groups showed discontinuous and fragmented expression of the tight junction proteins ZO-1 and occludin (red triangle), with loss of their characteristic continuous linear distribution **(**Figs. 3A-B**)**. Concurrently, expression of vimentin, a Sertoli cell cytoskeletal protein, was significantly downregulated **(**Figs. 3C**)**. RES intervention effectively rescued the expression and distribution of ZO-1, occludin, and vimentin, suggesting a restorative effect on BTB integrity. PS-NPs exposure also triggered testicular inflammation **(**Figs. 3D-E**)**. Expression of the pro-inflammatory cytokine TNF-α was significantly upregulated, whereas that of the anti-inflammatory cytokine IL-10 was downregulated, indicating a shift toward a pro-inflammatory state. Administration of RES effectively reversed these alterations, with TNF-α and IL-10 levels approaching normal.

### PS-NPs exposure triggered transcriptional alterations and enrichment in the PI3K-AKT pathway

**Fig. 4:**
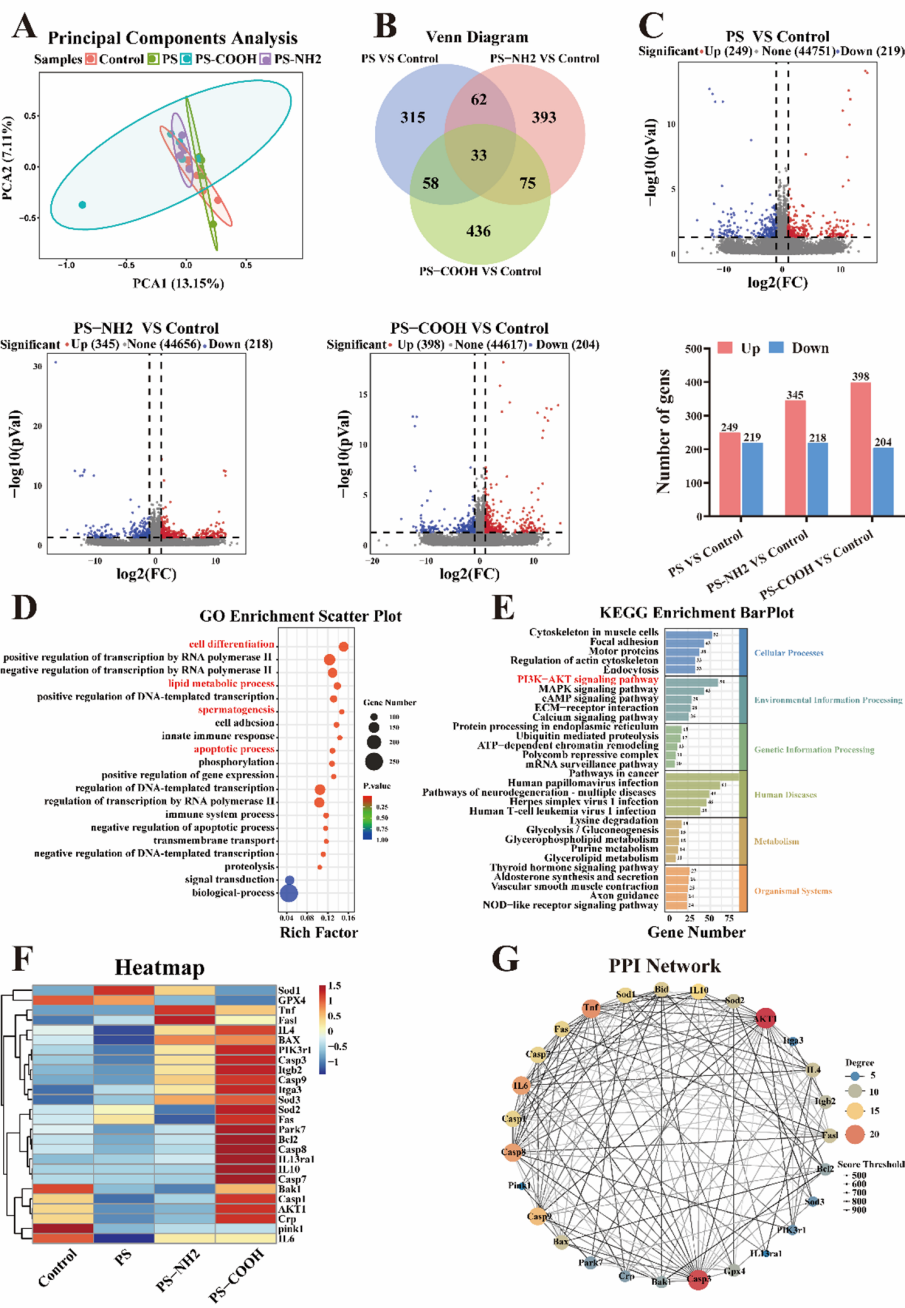
Comprehensive transcriptomic analysis revealed the impact of PS-NPs exposure on testicular tissue in ICR mice. (**A**) Principal component analysis (PCA) of the transcriptomes across all experimental groups. (**B**) Venn diagram illustrating the overlap of differentially expressed genes (DEGs) among the different comparison groups. (**C**) Volcano plot of DEGs. Red and blue dots represent significantly upregulated and downregulated genes, respectively, with bar charts summarizing the counts of up and downregulated genes for each comparison group. (**D**) Heatmap of DEGs significantly enriched in the PI3K-AKT signaling pathway. (**E**) GO enrichment analysis showing the top 20 most significantly enriched biological process terms. (**F**) KEGG pathway enrichment analysis bar chart. (**G**) Protein-protein interaction (PPI) network of the core DEGs. The node size and color intensity represent the degree of connectivity

Principal component analysis (PCA) revealed that the global perturbations induced by PS-NPs were distinct from the control and influenced by their surface chemistry **(**Fig. 4A**)**. A total of 33 DEGs were commonly altered in all PS-NPs exposure groups compared with the control **(**Fig. 4B**)**. Volcano plots identified 468, 563, and 602 significant DEGs in the PS, PS-NH₂, and PS-COOH groups, respectively **(**Fig. 4C**)**. To explore the biological implications of the DEGs, Gene Ontology (GO) and KEGG pathway enrichment analyses were performed. GO analysis revealed significant enrichment in key processes including RNA polymerase II-mediated transcription, cell differentiation, lipid metabolism, spermatogenesis, and apoptosis **(**Fig. 4D**)**. KEGG annotation classified the DEGs into major functional categories such as cellular processes, environmental information processing, and metabolism **(**Fig. 4E**)**. Among these, the PI3K-AKT signaling pathway was the most significantly enriched under the environmental information processing category. Given its established roles in inflammation and apoptosis, this pathway was selected for further investigation. We then generated a heatmap of core DEGs enriched in the PI3K-AKT pathway **(**Fig. 4F**)**, which clearly illustrated exposure-specific expression patterns. To further decipher the functional interplay among key elements, a protein-protein interaction (PPI) network was constructed by integrating PI3K-AKT pathway genes with those associated with inflammation and apoptosis **(**Fig. 4G**)**. This network-level analysis helped elucidate how the PI3K-AKT signaling axis may molecularly interface with inflammatory and apoptotic processes, thereby uncovering a core regulatory framework underlying microplastic toxicity.

### Effects of PS-NPs and RES on the expression of genes related to inflammation, apoptosis, and the PI3K-AKT pathway

**Fig. 5:**
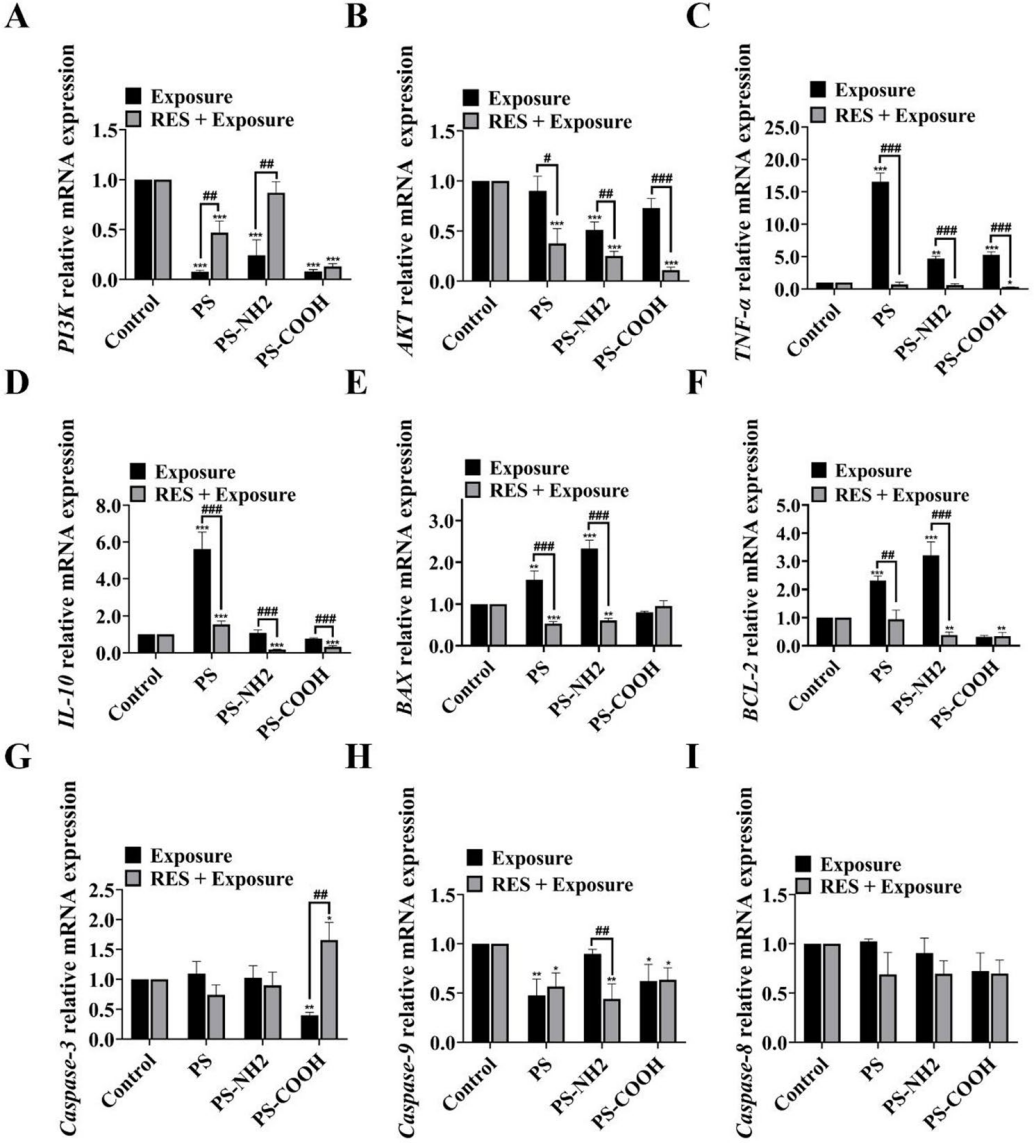
PS-NPs exposure altered the gene expression profiles related to the PI3K-AKT pathway, inflammation, and apoptosis. (**A-I**) Relative mRNA expression levels of key genes related to the PI3K-AKT signaling pathway, inflammatory responses, and apoptosis. Data are presented as the mean ± SD (*n* = 5); statistical significance is shown as follows: ^*^ for comparisons between the exposed group and the control group (^*^P < 0.05, ^**^P < 0.01, ^***^P < 0.001); ^#^ for comparisons between the exposed group and the antagonistic group (^#^*P* < 0.05, ^##^*P* < 0.01, ^###^*P* < 0.001)

To validate the transcriptomic findings, RT-qPCR was performed to examine the expression of key genes **(**Fig. 5**)**. Among inflammation-related genes, all PS-NPs exposure groups significantly upregulated mRNA levels of the pro-inflammatory factor *TNF-α* and the anti-inflammatory factor *IL-10*. RES intervention effectively restored the expression of these inflammation-related genes toward normal levels. Exposure to PS and PS-NH₂ upregulated both the pro-apoptotic gene *BAX* and the anti-apoptotic gene *BCL-2*, while PS-COOH downregulated both genes. Notably, all three types of PS-NPs (except PS-COOH) consistently enhanced the expression of the apoptosis executioner *caspase-3* and suppressed *caspase-9*. RES co-treatment effectively mitigated these apoptotic alterations, indicating its protective role against PS-NPs-induced apoptotic signaling. Regarding pathway genes, exposure to PS-NPs downregulated the expression of *PI3K* and *AKT*. RES intervention rescued *PI3K* expression, but failed to restore *AKT*, indicating a partial alleviation of the pathway disturbance.

### PS-NPs disrupted testicular metabolism possibly via glycolytic shift and antioxidant activation

**Fig. 6:**
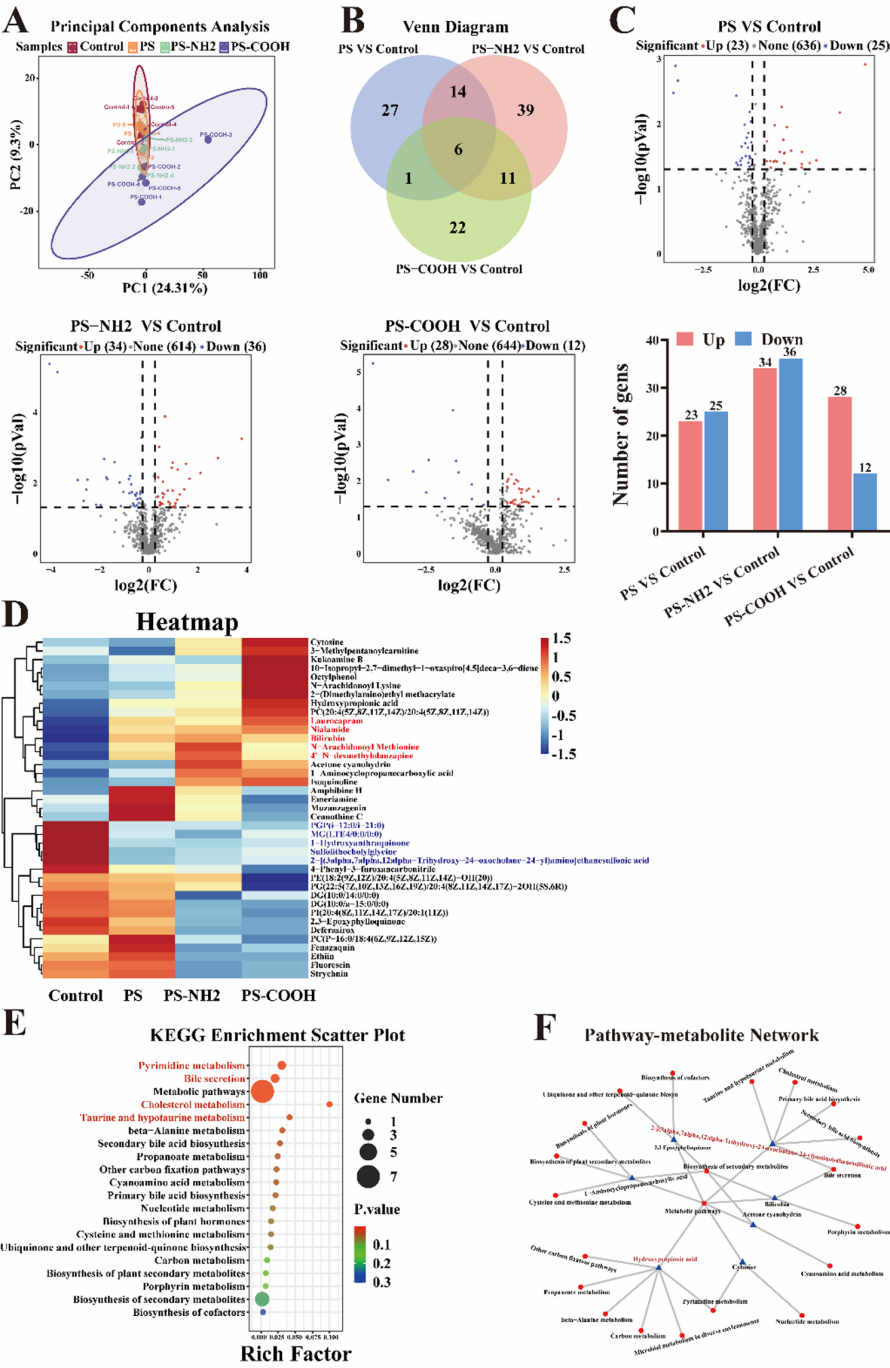
Testicular metabolic perturbations induced by PS-NPs exposure. (**A**) PLS-DA score plot reflecting differences between groups. (**B**) Venn diagram illustrating the overlap of differentially abundant metabolites (DAMs) among different comparison groups. (**C**) Volcano plot of DAMs. Red and blue dots represent significantly upregulated and downregulated metabolites, respectively. Bar charts summarize the counts of up and downregulated metabolites for each comparison group. (**D**) Heatmap showing the differentially expressed metabolites, with the samples along the horizontal axis and the metabolites along the vertical axis. (**E**) KEGG pathway enrichment bar chart. (**F**) KEGG pathway-metabolite interaction network. Triangles represent the top 30 most significant DAMs, and circles represent their enriched pathways

Metabolomic analysis revealed that PS-NPs exposure induced significant perturbations in the testicular metabolic profile of mice. Partial least squares-discriminant analysis (PLS-DA) showed clear separation among treatment groups **(**Fig. 6A**)**. Venn diagram analysis identified overlapping DAMs across comparison groups, with six metabolites consistently altered in all exposure groups, suggesting common mechanisms of toxicity **(**Fig. 6B**)**. Volcano plots visualized the overall distribution of DAMs, revealing 158 significantly altered metabolites versus controls (85 upregulated, 73 downregulated) **(**Fig. 6C**)**. The PS-NH₂ group exhibited the most pronounced metabolic disruption, with the highest number of DAMs (70 total), followed by the PS (48) and PS-COOH (40) groups. Heatmap analysis displayed distinct metabolite patterns across treatment groups **(**Fig. 6D**).** Five metabolites, including sulfolithocholylglycine, 2-[(3α,7α,12α-trihydroxy-24-oxocholane-24-yl)-amino] ethanesulfonic acid, PGP(i-12:0/i-21:0), MG(LTE4/0:0/0:0), and 1-hydroxyanthraquinone, were significantly downregulated in all exposed groups. Four additional metabolites, such as ethion, fluorescin, deferasirox, and PI (20:4(8Z,11Z,14Z,17Z)/20:1(11Z)), were downregulated specifically in both PS-NH₂ and PS-COOH groups. KEGG enrichment analysis associated the DAMs with pyrimidine metabolism, bile secretion, cholesterol metabolism, and taurine and hypotaurine metabolism **(**Fig. 6E**)**. Furthermore, a metabolite-pathway regulatory network identified lactic acid and taurine as hub molecules, participating in 7 and 6 metabolic pathways, respectively, indicating a shift toward glycolytic metabolism and activation of antioxidant defense mechanisms **(**Fig. 6F**)**.

### Combined analysis of transcriptomics and metabolomics

**Fig. 7:**
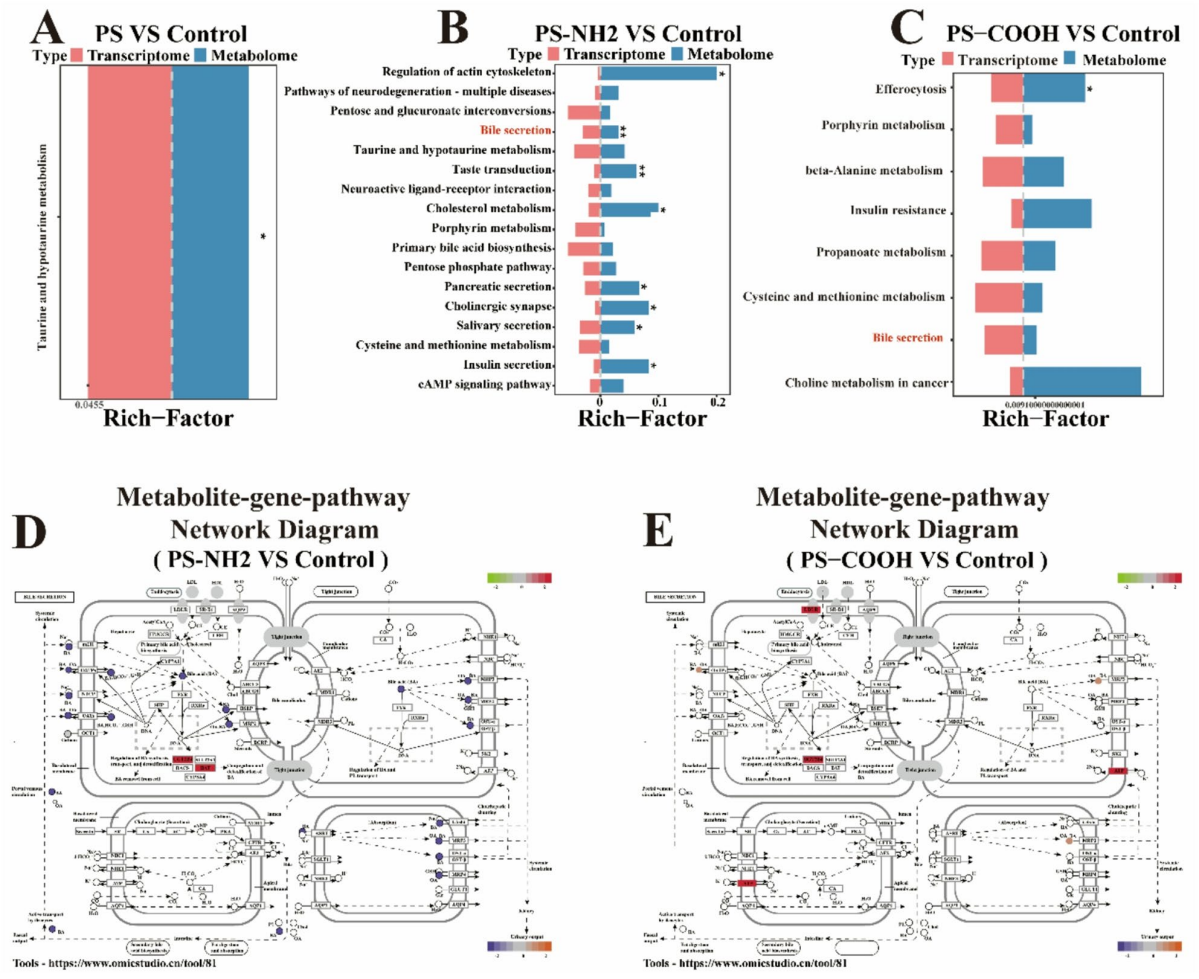
Multi-omics integration revealed key pathways and networks in PS-NPs-induced testicular toxicity. **(A-C**) Charts showing common pathways identified by transcriptomic and metabolomic analyses for the three exposure groups. Red represents differentially expressed genes (transcriptome), blue represents differentially expressed metabolites (metabolome), and the rich factor (calculated as the number of DAMs in the pathway divided by the total metabolites in that pathway) indicates the enrichment level (^*^P < 0.05). In (**D**) and (**E**), node colors represent expression/abundance levels: red (gene upregulation), green (gene downregulation), orange (metabolite upregulation), and purple (metabolite downregulation). (**D**) Network diagram of gene-metabolite interactions for PS-NH₂ vs. Control. (**E**) Network diagram of gene-metabolite interactions for PS-COOH vs. Control

Integrated analysis of transcriptomic and metabolomic data revealed pathway-level alterations common to different PS-NPs exposure groups, specifically, those pathways showing both significant differential gene expression and metabolite enrichment. In the PS-exposed group, taurine and hypotaurine metabolism was the primary shared pathway **(**Fig. 7A**)**. The PS-NH₂ group exhibited enrichment in multiple shared pathways, including actin cytoskeleton regulation, bile secretion, taste transduction, cholesterol metabolism, pancreatic secretion, insulin secretion, cholinergic synapse, and salivary secretion **(**Fig. 7B**)**. The PS-COOH group was primarily associated with shared pathways such as efferocytosis, insulin resistance, bile secretion, and choline metabolism in cancer **(**Fig. 7C**)**. Given that the bile secretion pathway was commonly enriched in both PS-NH₂ and PS-COOH groups, we selected it for further mechanistic investigation. To dissect its regulatory role under PS-NH₂ and PS-COOH exposure, a metabolite-gene interaction network within this pathway was constructed **(**Fig. 7D-E**)**. This network visually illustrates the coordinated relationships between differentially abundant metabolites and differentially expressed genes involved in bile secretion, providing insight into potential functional crosstalk at the molecular level.

#### RES ameliorated PS-NPs-induced apoptosis and PI3K-AKT activation

**Fig. 8:**
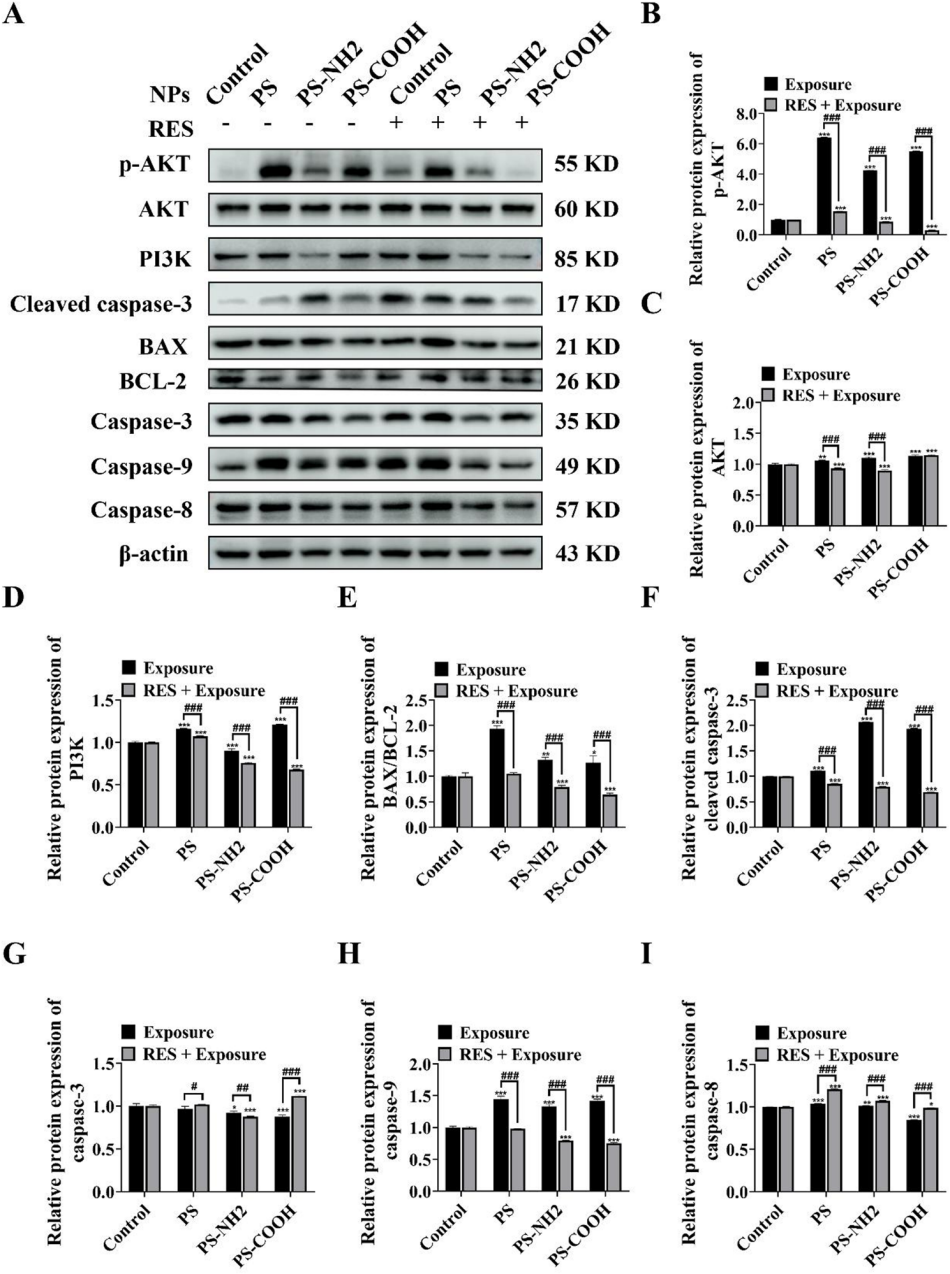
Effects of PS-NPs and RES on the PI3K-AKT signaling pathway and apoptosis-related proteins. (**A**) Western blot bands of p-AKT, AKT, PI3K, cleaved caspase-3, BAX, BCL-2, caspase-3, caspase-9, caspase-8, and β-actin. (**B-I**) Protein levels from the western blot analyses, normalized to β-actin. Data are presented as the mean ± SD (*n* = 5); statistical significance is indicated as follows: ^*^ for comparisons between the exposed group and the control group (^*^P < 0.05, ^**^P < 0.01, ^***^P < 0.001); ^#^ for comparisons between the exposed group and the antagonistic group (^#^*P* < 0.05, ^##^*P* < 0.01, ^###^*P* < 0.001)

To investigate the impact of PS-NPs exposure on the PI3K-AKT signaling pathway and apoptosis, and to evaluate the therapeutic potential of RES, we analyzed the expression of key associated proteins by western blot **(**Fig. 8A**)**. Compared to the control group, protein levels of PI3K and p-AKT were significantly upregulated in all PS-NPs-exposed groups, whereas RES co-treatment effectively suppressed this upregulation **(**Figs. 8B-C**)**. PS-NPs exposure markedly elevated the BAX/BCL-2 ratio **(**Fig. 8D**)** and enhanced the expression of cleaved caspase-3, the activated executioner protease of apoptosis **(**Fig. 8E**)**. Concurrently, the expression of caspase-9 was increased **(**Fig. 8F**)**, while caspase-8 expression showed no significant change or a slight increase **(**Fig. 8G). These results collectively indicate that PS-NPs may trigger the mitochondria-dependent intrinsic apoptotic pathway. Importantly, RES intervention significantly counteracted these pro-apoptotic alterations, demonstrating a clear anti-apoptotic function.

## Discussion

Polystyrene, a widely used and persistent plastic component, can be transformed into nanoplastics with diverse surface functional groups under environmental conditions, potentially exhibiting varied reproductive toxicities depending on their surface chemical properties [8]. Through integrated transcriptomic and metabolomic analyses, this study systematically demonstrates that PS-NPs, particularly functionalized PS-NPs, induce testicular dysfunction in mice by disrupting the blood-testis barrier, provoking inflammation and metabolic disturbances, and activating the PI3K-AKT signaling and apoptotic pathways. Furthermore, RES confers a significant protective effect against PS-NPs-induced damage.

In the present study, SEM confirmed the well-controlled, regular spherical morphology and uniform size distribution of PS, PS-NH₂, and PS-COOH particles, although their hydrodynamic diameters determined by DLS exceeded the manufacturer’s specifications, a finding consistent with the known tendency of DLS to report larger, intensity-weighted sizes due to the measurement of the hydrated particle radius and sensitivity to aggregates. Zeta potential analysis revealed negative surface charges for all three particle types, while Fourier-transform infrared spectroscopy verified the successful introduction of carboxyl and amine functional groups. These characterization results align with previous reports [32]. Notably, although amine groups can theoretically carry a positive charge, PS-NH₂ displayed the most negative zeta potential in deionized water. This observation may be attributed to the solution pH being below the pKa of the amine group, limiting protonation, combined with the inherent negative charge of the PS core and potential specific adsorption of anions at amine sites.

The testis serves as the primary site of spermatogenesis, where structural integrity and functional competence are essential for male fertility [33]. Our results showed that PS-NPs exposure induced spermatogenic cell detachment, reduced acrosomal cell counts, decreased sperm number, and increased sperm abnormality rates in mouse testes, indicating substantial impairment of testicular structure and function. These findings are similar to those reported in previous studies on testicular damage caused by varying sizes of PS-NPs [34]. RES intervention effectively mitigated the reduction in spermatogenic and sperm cells and lowered sperm abnormality, demonstrating its potential to alleviate PS-NPs-induced reproductive injury. This protective role is in agreement with a previous report that RES ameliorated bisphenol A-induced testicular damage, further supporting its utility in counteracting reproductive toxicity from environmental pollutants [23]. In comparing the toxicity of differently functionalized PS-NPs, this study revealed a noteworthy divergence from established literature. Previous studies generally indicate that positively charged PS-NH₂ exhibits greater bio-toxicity due to enhanced electrostatic adsorption to negatively charged cell membranes and subsequent internalization. PS-NH₂ induces stronger transgenerational reproductive toxicity in *Caenorhabditis elegans* than PS-COOH or PS at environmental concentrations [35]. Similarly, PS-NPs with functional group modifications demonstrate greater toxicity than their unmodified counterparts, inducing adverse effects such as oxidative stress, DNA damage, and testicular disruption [36]. In the current study, PS-COOH induced more severe spermatogenic impairment in our model. This suggests that beyond classical electrostatic adsorption-internalization routes, PS-COOH may employ alternative mechanisms to exert testicular toxicity. Studies have shown that nanoparticle surface properties strongly influence the composition of the acquired protein corona, and PS-COOH preferentially binds vitronectin with minimal conformational change and enters cells via integrin receptor-mediated endocytosis [37]. Thus, PS-COOH may be more efficiently internalized by testicular cells through such specific receptor-driven pathways, amplifying its toxic effects. This finding underscores that microplastic toxicity depends not only on surface charge but also on biomolecular recognition, protein corona formation, and subsequent internalization routes, providing a fresh perspective for assessing their health risks.

BTB is a specialized ultrastructure formed by tight junctions, adherens junctions, and gap junctions between adjacent Sertoli cells. It provides a highly secure microenvironment essential for germ cell differentiation and maturation by restricting the passage of blood-borne toxicants into the seminiferous tubule lumen [38]. Previous studies have shown that co-exposure to polystyrene and dibutyl phthalate resulted in a marked downregulation of the tight junction proteins ZO-1 and occludin [39]. In this study, exposure to all types of PS-NPs significantly downregulated the expression of ZO-1, occludin, and vimentin, indicating a compromised BTB. In contrast, RES treatment attenuated this damage by restoring their expression. Although no direct evidence has been reported on the effect of RES on PS-NPs-induced testicular toxicity, our data are consistent with its protective effect against reproductive dysfunction caused by other toxic pollutants [40]. This protective effect may be explained by its antioxidant and anti-cell death properties, as demonstrated in a previous study where RES ameliorates testicular toxicity associated with anti-PD-1 therapy by reversing BTB disruption [41].

Inflammatory activation represents a key mechanism underlying microplastic-induced testicular toxicity. In our study, PS-NPs exposure markedly disturbed testicular immune homeostasis, as evidenced by upregulation of the pro-inflammatory cytokine TNF-α. This imbalance indicates a shift toward a pro-inflammatory state in the testicular microenvironment, accompanied by insufficient endogenous anti-inflammatory capacity. These observations are consistent with previous reports on nanoparticle- or microplastic-induced reproductive toxicity. For instance, a prior study demonstrated that PS-MPs can suppress testosterone production by activating the macrophage-derived TNF-α/NF-κB signaling axis, which transcriptionally represses the *LHR* gene in Leydig cells [42]. Our results showed a decrease in IL‑10 levels detected by immunohistochemistry following PS‑NPs exposure, while the *IL‑10* gene expression was upregulated in exposed tissues. Immunohistochemistry provides cellular localization of the functional protein and is thus more directly related to the inflammatory status observed in the tissue. The observed reduction in IL‑10 protein is consistent with the overall pro‑inflammatory pattern, as evidenced by the elevated TNF‑α levels. The discrepancy between mRNA and protein levels is not uncommon in cytokine regulation and may reflect post-transcriptional control, differences in translational efficiency, or the composite nature of whole-tissue mRNA signals, which can mask cell-type specific protein changes. Several studies have reported that *IL‑10* gene expression is commonly suppressed upon exposure to pollutants such as airborne particulate matter and ammonia, consistent with a downregulation in its anti‑inflammatory function [43, 44]. However, under specific exposure contexts (e.g., dermal exposure to benzo[a]pyrene) or interventions (e.g., probiotic‑derived extracellular vesicles), the levels of *IL‑10* mRNA and protein can be increased [45, 46], highlighting the highly context‑ and tissue‑dependent regulation of IL‑10. This suggests that complex and environment‑dependent differences may exist between transcriptional and translational regulation of IL‑10, and further studies are needed to explain this phenomenon. Importantly, RES intervention counteracted the effects of PS-NPs, reversing the upregulation of TNF-α at both transcriptional and protein levels, and restoring the dysregulated expression of IL-10. This anti-inflammatory profile of RES is further corroborated by its efficacy in other reproductive disease models. For example, in rats with chronic prostatitis, RES treatment prominently reduced inflammatory cell infiltration, fibroblastic hyperplasia, and the expression of IL-6, IL-8, and TNF-α [47]. Collectively, our results suggest that PS-NPs exposure not only initiates inflammation but may also impair negative feedback mechanisms, leading to sustained and amplified inflammatory signaling and contributing to reproductive toxicity, which could be effectively reduced by RES.

To elucidate the underlying molecular mechanism, we performed RNA sequencing. Transcriptomic profiling revealed that PS-NPs exposure significantly altered the testicular gene expression landscape in mice, primarily involving biological processes such as apoptosis, lipid metabolism, and inflammation-related signaling. KEGG pathway analysis further identified the PI3K-AKT signaling pathway as the most significantly enriched core pathway, with the PPI network suggesting it as a key molecular hub linking PS-NPs exposure to inflammatory response and apoptotic activation. Our western blot validation confirmed that surface-functionalized PS-NPs promoted spermatogenic cell apoptosis and activated the PI3K-AKT pathway, which is consistent with previous findings that PS-NPs exposure induces activation of the PI3K-AKT-mTOR axis [36] However, the mRNA expressions of *PI3K* and *AKT* were downregulated by PS-NPs exposure. This likely represents a compensatory negative‑feedback mechanism. Excessive or sustained activation of the pathway proteins triggers intracellular feedback loops that suppress their own gene transcription in an attempt to restore cellular homeostasis. For instance, research on cadmium showed that p-AKT levels could be elevated without a change in total AKT protein, emphasizing the primacy of post-translational activation [48]. A study on vanadium pentoxide exposure documented a disconnection between PI3K subunit protein expression and phosphorylation states, demonstrating complex, non-linear regulation within this pathway [49]. These precedents confirm that PI3K-AKT pathway regulation is highly context-dependent, with mRNA levels often being a poor proxy for instantaneous pathway activity. In the present study, the apoptotic pathway was assessed by measuring the expression of apoptosis-related genes and proteins. Exposure to PS and PS-NH₂ upregulated both the pro-apoptotic gene *BAX* and the anti-apoptotic gene *BCL-2*, while PS-COOH downregulated both, suggesting that PS-NPs with different surface modifications differentially regulate the transcriptional levels of apoptosis-related genes. Despite the inconsistency in gene-level regulation, all three types of PS-NPs consistently enhanced the protein expression of the key apoptotic executioner protease caspase-3 and its activated form, cleaved caspase-3, which may be attributed to hierarchical signaling transduction or post-translational modifications in the regulatory pathway. Meanwhile, the increased expression of caspase-9 and the markedly elevated BAX/BCL-2 protein ratio collectively indicate that PS-NPs may primarily activate the mitochondria-dependent intrinsic apoptotic pathway. Both the mRNA level and protein expression of caspase-8 were also measured. The protein expression was increased by PS and PS-NH₂ but decreased by PS-COOH. In contrast, the mRNA level showed a similar trend of change across the three PS-NPs, but the differences were not statistically significant. Caspase-8 activation is a key event in initiating the extrinsic apoptotic pathway. This process involves the irreversible cleavage of the caspase-8 zymogen into its active form, which then propagates the death signal by cleaving downstream effectors like caspase-3. Under apoptotic stimuli, this rapid conversion, coupled with potential feedback mechanisms such as degradation of unused precursors via the ubiquitin-proteasome system (e.g., through formation of degradable heterodimers with proteins like c-FLIP(L)), can lead to complex net changes in total protein levels [50]. The decrease observed with PS-COOH might reflect such enhanced consumption and turnover, whereas the increase with PS and PS-NH₂ could indicate an initial upregulation of the zymogen pool. Caspase-8 transcription is known to be regulated by complex networks involving factors like NF-κB or p53 [51]. The different exposure to PS-NPs may subtly influence these upstream signaling pathways, leading to modest transcriptional perturbations that do not reach statistical significance in our assay. The disconnect between slight mRNA trends and significant protein changes underscores the biological complexity of gene expression regulation, highlighting that transcriptional levels alone are often insufficient predictors of protein abundance. Further research is needed to elucidate this relationship. Caspase-9 is the initiator caspase in the intrinsic apoptotic pathway. Its activation primarily occurs via post-translational cleavage and apoptosome formation, not directly through transcriptional upregulation. The mRNA level does not necessarily reflect the rapid, proteolytic activation of existing pro-caspase-9 protein pools in response to apoptotic stimuli. For instance, one study reported increased cleaved caspase-9 protein concurrent with decreased full-length caspase-9 expression under exposure to biomass combustion soluble constituents (BCSCs), highlighting activation through processing [52]. Conversely, studies on pollutants such as cadmium report coordinated increases in both caspase-9 mRNA and protein levels during reproductive toxicity induction [53], whereas studies on BPA exposure observe decreases in both mRNA and protein levels in ototoxicity models [54]. Therefore, the downregulation of *caspase-9* mRNA in our study may represent a distinct cellular response to PS-NPs exposure, potentially involving feedback inhibition or a shift in apoptotic regulation timing. In contrast, the increase in caspase-9 protein likely indicates the activation of the intrinsic apoptotic pathway through post-translational mechanisms, as supported by the markedly elevated BAX/BCL-2 protein ratio. Finally, RES intervention significantly counteracted these pro-apoptotic alterations, demonstrating its anti-apoptotic role by targeting key nodes in this pathway. Together, these findings reveal the complex regulatory network of PS-NPs with different surface chemistries in inducing apoptosis, wherein the incomplete concordance between gene and protein expression, as well as the differential responses across molecular levels, may represent critical directions for future mechanistic studies.

Furthermore, metabolomic analysis through KEGG enrichment revealed that PS-NPs exposure perturbed several key metabolic pathways in mouse testes, including pyrimidine metabolism, bile secretion, cholesterol metabolism, and taurine and hypotaurine metabolism. A metabolite-centric regulatory network highlighted lactic acid and taurine as multifunctional nodes, participating in 7 and 6 metabolic pathways, respectively, suggesting a systemic shift in energy metabolism and activation of endogenous antioxidant defenses. Specifically, perturbations in pyrimidine metabolism combined with elevated lactic acid levels indicate a metabolic rewiring from oxidative phosphorylation toward anaerobic glycolysis, a potential adaptive response to environmental stress. However, sustained glycolytic activation may compromise ATP production necessary for spermatogenesis, ultimately impairing sperm development. Similarly, a prior study showed that PS-NPs can interfere with lactate dehydrogenase (LDH) activity, thereby disrupting glycolytic flux [55]. Altered taurine metabolism implies mobilization of intrinsic cytoprotective mechanisms against PS-NPs-triggered oxidative stress. Taurine, a sulfur-containing amino acid with recognized antioxidant and membrane-stabilizing functions, has been shown to enhance germ cell quality by preserving mitochondrial integrity, attenuating ROS generation, and restoring cytoskeletal architecture, which correlates with the pathway enrichment trends previously identified in our sequencing data [56]. Of particular interest is the disruption of cholesterol metabolism. As the precursor for steroid hormone biosynthesis, dysregulated cholesterol homeostasis may directly impede testosterone synthesis. Prior work has demonstrated that high-cholesterol diets suppress testosterone production in rat Leydig cells [57]. Our results imply that PS-NPs interfere not only with spermatogenesis but may also exert endocrine-disrupting effects, thereby expanding the mechanistic understanding of their reproductive toxicity from a metabolic perspective.

Integrated analysis of DEGs and metabolites revealed that PS-NPs with different surface chemistries exert toxicity through disruption of distinct biological pathways. Notably, both PS-NH₂ and PS-COOH exposure perturbed metabolic pathways related to bile secretion, suggesting that their toxic effects may extend beyond local testicular injury to involve systemic physiological disturbances. Based on these observations, we propose that PS-NPs may impair testicular function indirectly via a liver-testis axis. This hypothesis aligns with established reports of other environmental pollutants affecting reproductive function through liver-mediated interorgan pathways. For instance, it has been demonstrated that 4-methylbenzylidene camphor elicits estrogenic effects via a brain-liver-gonad axis [58], and that perfluorooctane sulfonate disrupts endocrine activity and impairs reproduction in male zebrafish through a hypothalamus-pituitary-gonad-liver axis [59]. Although these axes differ in their specific components, they collectively underscore the liver’s role as a metabolic hub in mediating pollutant-induced reproductive toxicity. Our findings further support this concept, suggesting that PS-NPs may disrupt hepatic bile acid metabolism, thereby systemically influencing testicular cholesterol homeostasis and hormone synthesis through circulating factors, ultimately contributing to reproductive endocrine dysfunction via the liver-testis axis.

While this multi-omics study provides comprehensive insights into the testicular toxicity of differentially functionalized PS-NPs and the protective mechanisms of RES, several limitations should be acknowledged. First, although the administered dose (50 mg/kg/day) was derived from estimated human intake levels, the results must be interpreted with caution in assessing real-world risks. Future studies should incorporate lower levels and long-term exposure to improve risk assessment accuracy. Second, although we observed concurrent disruption of bile secretion and activation of the PI3K-AKT-mediated inflammatory and apoptotic pathways, the causal and regulatory relationships between these phenomena remain unclear and warrant further investigation. Finally, this study examined only amine- and carboxyl-modified PS-NPs; however, environmental microplastics exhibit far greater surface complexity and diversity (e.g., aged PS-NPs or co-exposure with other pollutants), which may lead to a broader spectrum of toxicological profiles and therefore warrants further investigation. In summary, future work should focus on delineating the interactions between identified metabolic and transcriptional networks to construct a more comprehensive mechanistic map of PS-NPs-induced reproductive toxicity.

## Conclusions

This study systematically investigates the male reproductive toxicity of PS-NPs with different surface functionalizations and elucidates the underlying molecular mechanisms. PS-NPs exposure induces structural disruption of testicular tissue, compromised blood-testis barrier integrity, reduced sperm count, and increased sperm abnormality. Notably, functionalized PS-NPs exhibit greater reproductive toxicity among the tested particles. Integrated transcriptomic and metabolomic analyses reveals that PS-NPs may trigger testicular inflammation, spermatogenic cell apoptosis, and activation of the PI3K-AKT signaling pathway, while also perturbing energy metabolism and endogenous antioxidant defense systems, collectively contributing to reproductive impairment. Furthermore, the bile secretion pathway was identified as a common metabolic node disrupted by both PS-NH₂ and PS-COOH exposure, suggesting its potential key role in mediating PS-NPs reproductive toxicity, a finding that merits further investigation into its regulatory mechanisms. In terms of intervention, the natural antioxidant RES effectively mitigates PS-NPs-induced inflammation and apoptosis and improved sperm-related parameters, demonstrating its potential as a protective agent against microplastic-induced reproductive damage.

## Supplementary Information

Supplementary Material 1

## Data Availability

Data will be made available on request.

## Abbreviations

MNPs: Micro - and nanoplastics
NPs: Nanoplastics
PS-NPs: Polystyrene nanoparticles
PS: Polystyrene
PS-NH₂: Amino-modified PS-NPs
PS-COOH: Carboxyl-modified PS-NPs
RES: Resveratrol
ZO-1: Zonula occludens-1
TNF-α: Tumor necrosis factor-alpha
IL-10: Interleukin-10
PI3K: Phosphatidylinositol 3-kinase
AKT: AKT serine/threonine kinase
BAX: BCL2-associated X protein
BCL-2: B-cell lymphoma 2
Caspase-3: Cysteine aspartic acid-specific protease-3
Caspase-8: Cysteine aspartic acid-specific protease-8
Caspase-9: Cysteine aspartic acid-specific protease-9
GO: Gene ontology
KEGG: Kyoto encyclopedia of genes and genomes
DEGs: Differentially expressed genes
DAMs: Differentially abundant metabolites
QC: Quality control
