# Supplementary material for: Transcriptomics and metabolomics reveal functional nanoplastics-induced male reproductive damage and resveratrol antagonistic effects
Source: J Nanobiotechnology. 2026 Mar 16;24:386. doi: 10.1186/s12951-026-04283-8 (PMC13104249; doi:10.1186/s12951-026-04283-8)
Supplement: Supplementary file 1 — Supplementary Material 1 [file 12951_2026_4283_MOESM1_ESM.docx]

**Transcriptomics and metabolomics reveal functional nanoplastics-induced male reproductive damage and resveratrol antagonistic effects**

Fang Zhang^1*^, Nenghua Zhang^2*^, Chunji Wang ^1,3^, Li Zhang ^4^, Yujia Yang ^4^, Yue Jia^1^, Xiaowen Huang ^2^, Minghui Li ^5^, Jie Tang ^2#^, Long Xu ^1#^

^1^ College of Biological, Chemical Science and Engineering, Forensic and Pathology Laboratory, College of Medicine, Jiaxing University, Jiaxing 314001, ZJ, China

^2^ Central Laboratory of Jiaxing Hospital of Traditional Chinese Medicine, Jiaxing University, Jiaxing 314001, ZJ, China

^3^ College of Life Sciences and Medicine, Zhejiang Sci-Tech University, Hangzhou 310053, ZJ, China

^4^ School of Life Sciences, Zhejiang Chinese Medical University, Hangzhou 310053, ZJ, China

^5^ School of Bioengineering, Chongqing University, Chongqing 400045, China

* These authors contributed to this work equally.

# Author to whom correspondence should be addressed.

Long Xu: xl_st2011@outlook.com

Jie Tang: tjie1993@163.com

Tel: +86 13484110307

Fax: +86 573 83640031


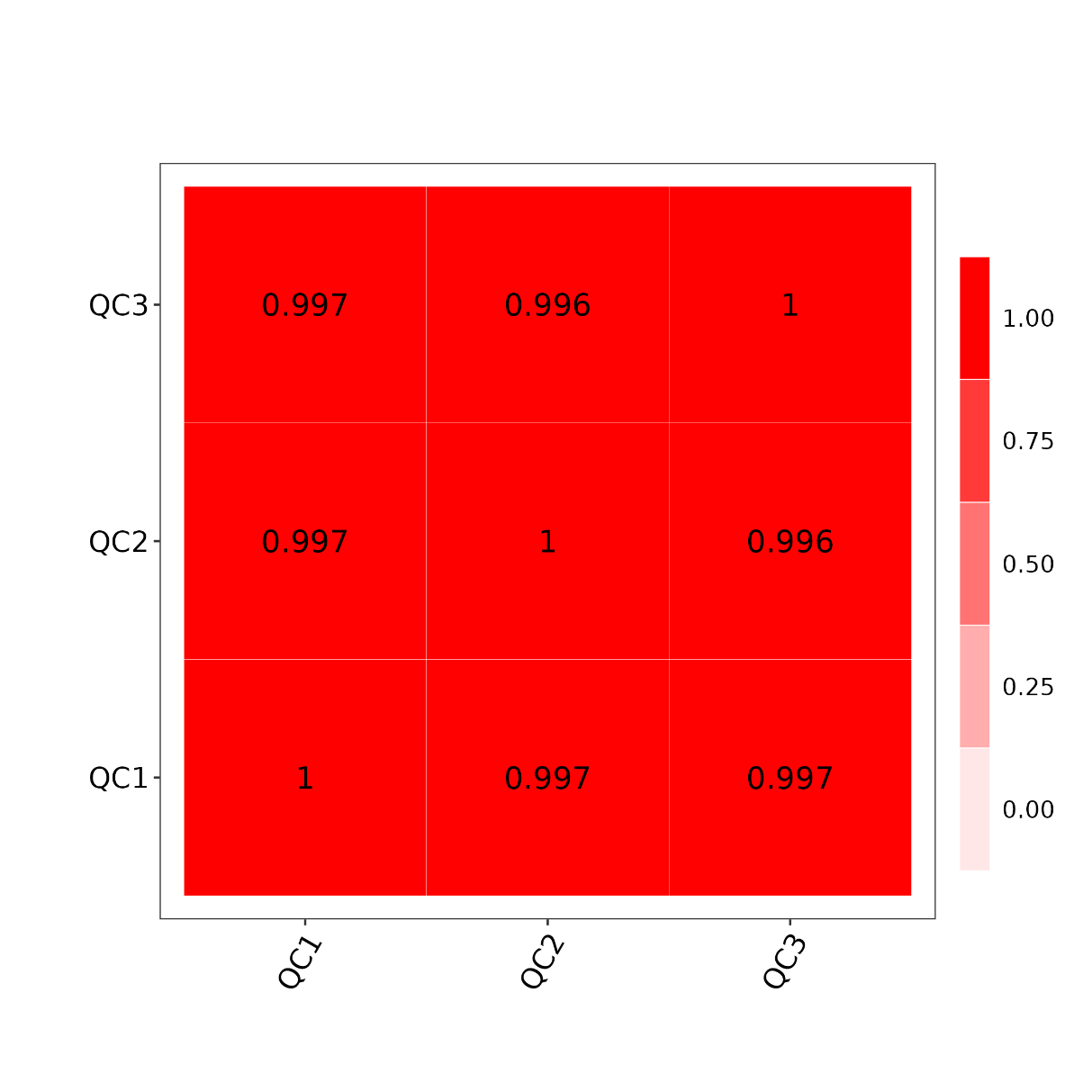


**Supplementary Figure 1** Heatmap of Pearson correlation coefficients among quality Control samples.

**Supplementary Table 1** Primers of the genes used in the study.

| **Gene** | **Forward** | **Reverse** |
| --- | --- | --- |
| *PI3K* | ACACCACGGTTTGGACTATGG | GGCTACAGTAGTGGGCTTGG |
| *AKT* | ATGAACGACGTAGCCATTGTG | TTGTAGCCAATAAAGGTGCCAT |
| *TNF-α* | CCCTCACACTCAGATCATCTTCT | GCTACGACGTGGGCTACAG |
| *IL-10* | GCTCTTACTGACTGGCATGAG | CGCAGCTCTAGGAGCATGTG |
| *BAX* | TGAAGACAGGGGCCTTTTTG | AATTCGCCGGAGACACTCG |
| *BCL-2* | GTCGCTACCGTCGTGACTTC | CAGACATGCACCTACCCAGC |
| *Caspase-3* | TGGTGATGAAGGGGTCATTTATG | TTCGGCTTTCCAGTCAGACTC |
| *Caspase-9* | TCCTGGTACATCGAGACCTTG | AAGTCCCTTTCGCAGAAACAG |
| *Caspase-8* | TGCTTGGACTACATCCCACAC | TGCAGTCTAGGAAGTTGACCA |
| *β-actin* | GGCTGTATTCCCCTCCATCG | CCAGTTGGTAACAATGCCATGT |

**Supplementary Note 1** Chromatographic separation and high-resolution mass spectrometric detection methods.

The liquid chromatography component consisted of an ACQUITY UPLC system (Waters, Milford, MA, USA) equipped with a Kinetex UPLC C18 column (100 mm × 2.1 mm, 100 A; Phenomenex, UK) maintained at 55°C. The mobile phases were A (acetonitrile: water = 6:4, containing 0.1% formic acid) and B (isopropanol: acetonitrile = 9:1, containing 0.1% formic acid). The flow rate was 0.3 mL/min, and the gradient elution program was as follows: 0-0.4 min, 30% B; 0.4-1 min, 30% → 45% B; 1-3 min, 45% → 60% B; 3.5-5 min, 60% → 75% B; 5-7 min, 75% → 90% B; 7-8.5 min, 90% → 100% B; 8.5-8.6 min, 100% B; 8.6-8.61 min, 100% → 30% B; 8.61-10 min, 30% B. Mass spectrometry detection was carried out on a TripleTOF 6600 high‑resolution tandem mass spectrometer (SCIEX, Framingham, MA, USA). Data were acquired in both positive and negative ionization modes. The ion source parameters were: Curtain Gas 30 PSI, Ion Source Gas 1 and Gas 2 both 60 PSI, Interface Heater Temperature 650°C. The ion spray voltage was set to +5000 V for positive mode and -4500 V for negative mode. Information‑dependent acquisition (IDA) was employed with a TOF scan range of 60-1200 Da and a Survey Scan time of 150 ms. Product‑ion scans were triggered when the signal intensity exceeded 100 cps with a charge state of 1+, and up to 12 MS² spectra were collected per cycle (cycle time 0.56 s). A 40‑GHz multi‑channel TDC detector (four anodes/channel) with a pulse frequency of 11 kHz was used, and dynamic exclusion was set to 4 s.
